# Real-World Outcomes of Ribociclib Treatment in Patients With Metastatic Breast Cancer at a Tertiary Care Hospital

**DOI:** 10.7759/cureus.84418

**Published:** 2025-05-19

**Authors:** Shakeel Muzaffar, Mussadique Ali Jhatial, Naqib Ullah, Muhammad Awais Majeed, Muhammad Qasim, Muhammad Bilal Rasheed, Tahira Yasmeen, Neelam Siddiqui

**Affiliations:** 1 Medical Oncology, Shaukat Khanum Memorial Cancer Hospital and Research Centre, Lahore, PAK; 2 Medical Oncology, King Faisal Specialist Hospital and Research Centre, Riyadh, SAU

**Keywords:** hr+ve/her2-ve breast cancer, metastatic breast cancer, outcomes, ribociclib, toxicity

## Abstract

Background

Ribociclib is an important treatment option in patients with hormone receptor-positive (HR+) HER2-negative (HER2-) advanced/metastatic breast cancer (ABC/MBC); however, there are limited data on its efficacy and safety in the South Asian population. This study aims to evaluate outcomes of breast cancer patients treated with ribociclib therapy in terms of progression-free survival (PFS), overall survival (OS), overall response rate (ORR), duration of response (DOR), and assessment of frequency and severity of toxicity at a tertiary care setup in a resource-limited setting.

Methodology

This was a single-center, retrospective observational study. Patients aged 18 years and older, diagnosed with HR+ HER2- ABC/MBC, registered between January 2016 and December 2021, and treated with ribociclib therapy were included in this study.

Results

Median PFS for the overall cohort (n = 50) was 14.7 months, with 10 (20%) patients having no progression on last follow-up. Median OS was 30.8 months, and 19 (38%) patients were alive at the time of last follow-up. Median PFS and OS were significantly longer in patients with non-visceral metastatic disease than with extracranial visceral and brain metastatic disease. The ORR was 48%, whereas the clinical benefit rate was 76%, and the median DOR was 18.9 months. Eleven patients achieved a complete response during the course of their treatment. The most common adverse effect was neutropenia (68%), which was manageable with appropriate dose reductions.

Conclusion

Our retrospective data support the notion that ribociclib-based therapy is effective and safe in patients with HR+/HER2- ABC/MBC; however, as it is a retrospective study with a small sample size, further prospective studies are necessary to further prove these results in the local population, and efforts should be taken in society at large to make this treatment available to all patients who require this treatment.

## Introduction

Breast cancer is the second most commonly diagnosed malignancy (11.6%) and the fourth most common cause of cancer-related mortality (6.9%) in both genders combined globally. In females, it is the most commonly diagnosed cancer (23.8%) and the most common cause of cancer-related mortality (15.4%) [1]. In women under the age of 50, approximately 26.5% of breast cancer cases are hormone-receptor-positive (HR+)/human epidermal growth factor receptor 2-negative (HER2−) [2], whereas the incidence of this subtype is 70% when all age groups are considered [3]. 

For women with HR+/HER2- advanced or metastatic breast cancer (ABC/MBC), endocrine therapy (ET) has traditionally been the treatment of choice except for patients with visceral crisis [4]. However, despite these treatments, many patients eventually develop resistance to ET, leading to disease progression [4]. 

Endocrine resistance is classified into two types: primary resistance, where patients experience disease progression during the first six months of first-line ET for ABC/MBC, and secondary resistance, which is defined as disease progression after six months of ET in the metastatic setting [5]. 

Cancer progression is primarily driven by the uncontrolled proliferation of cells due to cell cycle dysregulation [6]. One of the mechanisms driving this uncontrollable proliferation is dysregulation of the cyclin-CDK-E2F-Rb pathway, which can be secondary to (a) overexpression of cyclin D1 (CD1) or (b) loss of endogenous CDK inhibition due to (i) absence of p16INK4a, (ii) mutation of CDK4 to a p16INK4a-refractory state, or (iii) loss of the Rb1 gene [7]. 

Inhibition of this pathway and prevention of the phosphorylation of the Rb gene lead to cell-cycle arrest by halting the cell cycle in the G1 phase. Cyclin-dependent kinase 4/6 (CDK4/6) inhibitors, such as ribociclib, prevent phosphorylation of Rb protein and arrest the cell cycle in the G1 phase [8]. They have thus become an essential treatment option for HR+/HER2− ABC/MBC by targeting this mechanism. These inhibitors work in combination with ET to help overcome resistance and improve patient outcomes [9]. 

Ribociclib is one of the three FDA-approved CDK4/6 inhibitors, with palbociclib and abemaciclib [10]. Clinical trials have demonstrated their effectiveness in improving survival rates with respect to progression-free survival (PFS) and overall survival (OS). The phase III Mammary ONcology Assessment of LEE011's Efficacy and SAfety-2 (MONALEESA-2) trial showed that combining ribociclib with letrozole resulted in a median PFS of 25.3 months compared with 16.0 months with letrozole alone (HR: 0.57; 95% CI: 0.46-0.69; P < 0.001) [11]. Updated results from this trial indicated a median OS of 63.9 months [12]. Similarly, the MONALEESA-7 trial, which focused on premenopausal patients, reported a significant improvement in OS when ribociclib was combined with ET [13]. 

Although ribociclib has shown promising results in Western populations, there are limited data on its efficacy in developing regions such as South Asia due to limited availability, limited patient affordability, and scarcity of prospective data. This study aims to evaluate the outcomes of ribociclib therapy in terms of PFS, OS, response rates, and treatment-related toxicity in metastatic breast cancer patients in our local population. Understanding these outcomes will provide valuable insights to help optimize treatment strategies and improve patient care in resource-limited settings. 

## Materials and methods

Study design and participants 

This is a retrospective study designed to evaluate PFS and OS and the association of patient and disease characteristics with PFS and OS of HR+/HER2- ABC/MBC treated with ribociclib therapy. The Institutional Review Board of Shaukat Khanum Memorial Cancer Hospital & Research Center (SKMCH&RC), Lahore, Pakistan, approved this as a retrospective study and granted a waiver of informed consent. For the collection of data, the hospital information system (HIS) of our institute was accessed, using a keyword search for breast cancer and ribociclib. A total of 67 patients were identified, but only 50 patients met the study inclusion criteria. 

Eligible patients were those with HR+/HER2- ABC/MBC, diagnosed by histopathology and treated with ribociclib therapy for a minimum of four months at SKMCH&RC between January 01, 2016, and December 31, 2021. A sample size of 67 was identified based on the WHO sample size calculator with a population proportion of 4.5% (given the ratio of patients with early versus metastatic breast cancer patients registered in the hospital during the study period), the allowed margin of error was 5%, and the confidence interval was 95%. Patients having breaks or irregularities in treatment, those lost to follow-up, and those having incomplete essential data required for survival analysis were excluded from the study, bringing the sample size down to 50. 

Methods 

Patients’ medical records present in the electronic hospital information system were accessed to obtain data on patients’ demographics and disease characteristics. Electronic reports and physician notes were accessed to confirm histopathology, receptor status, disease stage, previous treatments received, date of starting ribociclib therapy and duration of treatment, combination hormonal agent received, and status at end of therapy and at last follow-up. 

We defined PFS and OS as the primary outcomes of our study and extent and duration of response (DOR), toxicity, and subgroup analysis of the effect of various patient and disease factors on PFS and OS as secondary outcomes. 

OS was assessed based on the interval duration between the date of starting ribociclib and the date of last follow-up and patient status at the last follow-up (alive or dead). Patients who were alive till the end of data collection (December 1, 2024) were considered censored in the survival analysis.

PFS was assessed by the interval between the dates of the start and end of ribociclib in cases of patients with progressive disease at the end of therapy or the dates between starting treatment and last follow-up with the patient if there was no documented disease progression. Those patients who had no radiological evidence of progression were censored in PFS till the date of last follow-up (December 1, 2024).

The objective response rate was calculated as the percentage of patients who had partial or complete responses based on Response Evaluation Criteria in Solid Tumors (RECIST) criteria on the first follow-up scan after commencing therapy [14]. Duration of response was assessed by the interval duration between the date of response and the date of progression. Treatment-related toxic side effects were graded according to the Common Terminology Criteria for Adverse Events (CTCAE) grading system, and their frequency was calculated accordingly. 

Statistical analysis 

The data were tabulated and analyzed using IBM SPSS Statistics software for Microsoft Windows, version 26.0 (IBM Corp., Armonk, NY). Age was described by median and range. Frequency was tabulated as a percentage by gender, menopausal status, histopathology, and receptor status, disease status, clinical presentation, whether previously treated or treatment-naïve, metastatic sites, prior treatment used in adjuvant and metastatic settings, and type of endocrine agent taken in combination with ribociclib. 

The Kaplan-Meier method was used to calculate survival analysis, and P-values were calculated using the log-rank test; a p-value < 0.05 was considered significant. 

Survival was compared with respect to histopathology, presentation, whether previously treated or naïve, metastatic sites, prior treatments received in adjuvant and metastatic settings, and type of endocrine agent taken in combination with ribociclib. Subgroup analysis was conducted in order to determine the effect of the “above-mentioned” factors/confounders on patients’ survival. Treatment responses were documented as per standard RECIST as complete response (CR), partial response (PR), stable disease (SD), or progressive disease (PD). 

## Results

The study included 50 patients. Age at presentation ranged from 31 to 72 years (median = 52.50); 49 patients were female, and one patient was male; 30 (60%) were postmenopausal, and 20 (40%) were premenopausal. Of 50, 27 (54%) had de novo metastatic disease and hence were treatment-naïve, whereas 23 (46%) had disease recurrence with distant metastases. On histopathology, 31 (62%) had invasive ductal carcinoma (IDC) Grade II, 14 (28%) had IDC Grade III, three (6%) had invasive lobular carcinoma (ILC) Grade II, and two (4%) had ILC Grade III disease. 

With regard to metastatic sites, 27 (54%) patients had visceral metastatic disease, 11 (22%) had bone and distant nodal metastases, five (10%) had brain metastases along with extracranial visceral disease, five (10%) had only bone disease, and two (4%) had only lymph node (LN) disease. Thirty-one patients (62%) had not received any treatment in a metastatic setting, six (12%) had ET, 10 (20%) had both ET and chemotherapy, and three (6%) had chemotherapy only. Thirty patients (60%) received letrozole, 12 (24%) received fulvestrant, six (12%) received tamoxifen, and two (4%) received exemestane in combination with ribociclib. Thirty-one (62%) patients had died by the time of the last follow-up during this study. Demographic and clinical characteristics of patients are shown below in Table 1.

**Table 1:** Demographic and clinical characteristics of patients IDC: invasive ductal carcinoma; LN: lymph node; ET: endocrine therapy

Parameters	N (%)
All patients	50
Gender	
Female	49
Male	1
Menopausal status	
Premenopausal	19 (38)
Postmenopausal	30 (60)
Metastatic presentation	
Denovo / treatment-naive	27 (54)
Recurrent/previously treated	23 (46)
Histopathology	
IDC Grade II	31 (62)
IDC Grade III	14 (28)
ILC Grade II	03 (6)
ILC Grade III	02 (4)
Metastatic sites	
Bones only	05 (10)
LN only	02 (4)
Distant nodal and bones	11 (22)
Visceral metastases	27 (54)
Brain and extracranial visceral metastases	5 (10)
Prior treatment	
Adjuvant setting	
None (de novo metastases)	27 (56)
ET only	12 (24)
ET+chemotherapy	11 (22)
Metastatic setting	
None	31 (62)
ET only	06 (12)
ET+chemotherapy	10 (20)
Only chemotherapy	03 (6)
Combination agent	
Tamoxifen	06 (12)
Letrozole	30 (60)
Fulvestrant	12 (24)
Exemestane	2 (04)

Primary outcomes 

PFS

**Figure 1:**
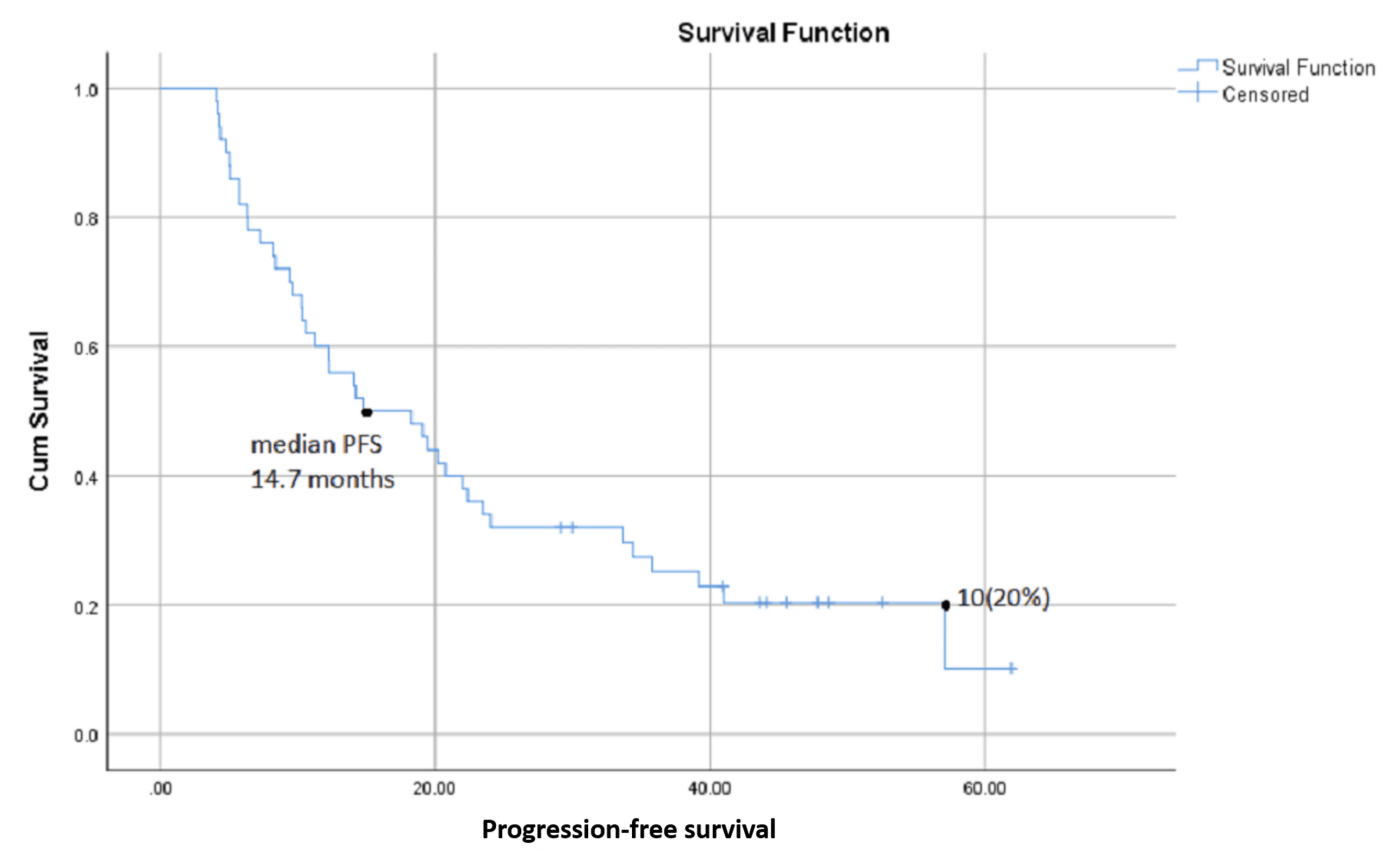
Progression-free survival (PFS) in months from commencement of ribociclib therapy

Median PFS for the overall cohort (n = 50) was 14.7 months, with 10 (20%) patients having no progression at the time of last follow-up during this study (Figure 1). 

OS

**Figure 2:**
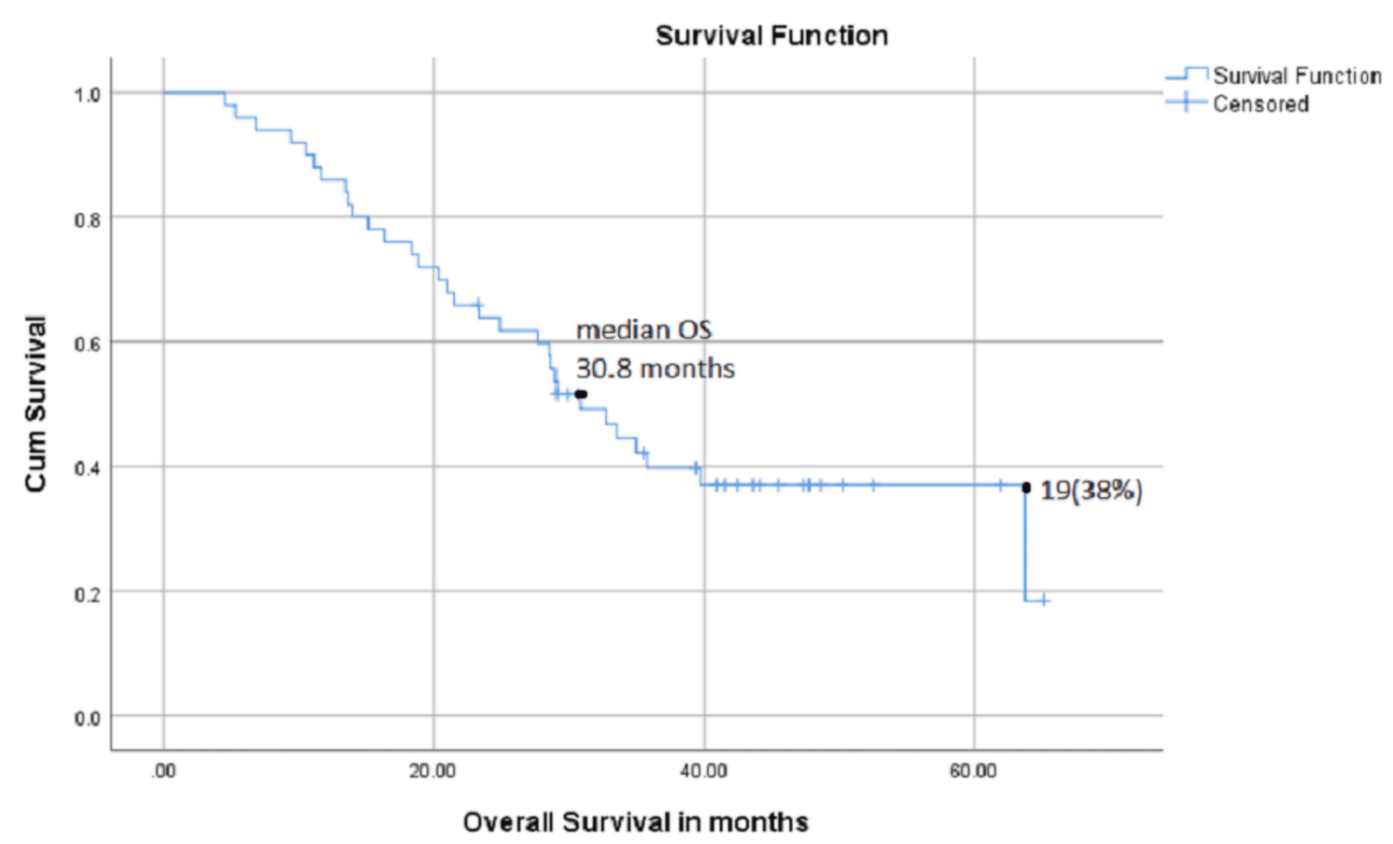
Overall survival (OS) in months from the commencement of therapy to date of death or last follow-up.

Median OS was 30.8 months in the overall study population, and 19 patients were alive at the time of last follow-up during this study (Figure 2). 

Secondary outcomes 

Response assessment scans performed at four months from commencement of therapy demonstrated complete response in nine (18%) patients; 15 (30%) had a partial response, 14 (28%) had stable disease, and 12 (24%) had progressive disease. The overall response rate (ORR) was 48%, the clinical benefit rate was 76%, and 11 patients achieved complete response during the course of their treatment. 

In the cohort that responded to treatment, the median duration of response was 18.9 months. Patients who were treatment-naïve (n = 27) had a median DOR of 29.8 months. DOR was 8.9 months for those who presented with recurrent metastatic disease and had been treated previously (n = 23). DOR also varied with the type of treatment received prior to ribociclib in a metastatic setting (P-value 0.014). Treatment-naive patients (n = 25) had a median DOR of 29.8 months; those who had ET (n = 6) had a median DOR of 8.6 months. Patients who had received ET as well as chemotherapy (n = 4) had a DOR of 15.3 months, and DOR was only 4.4 months in the group that had only received chemotherapy previously (n = 2). 

**Figure 3:**
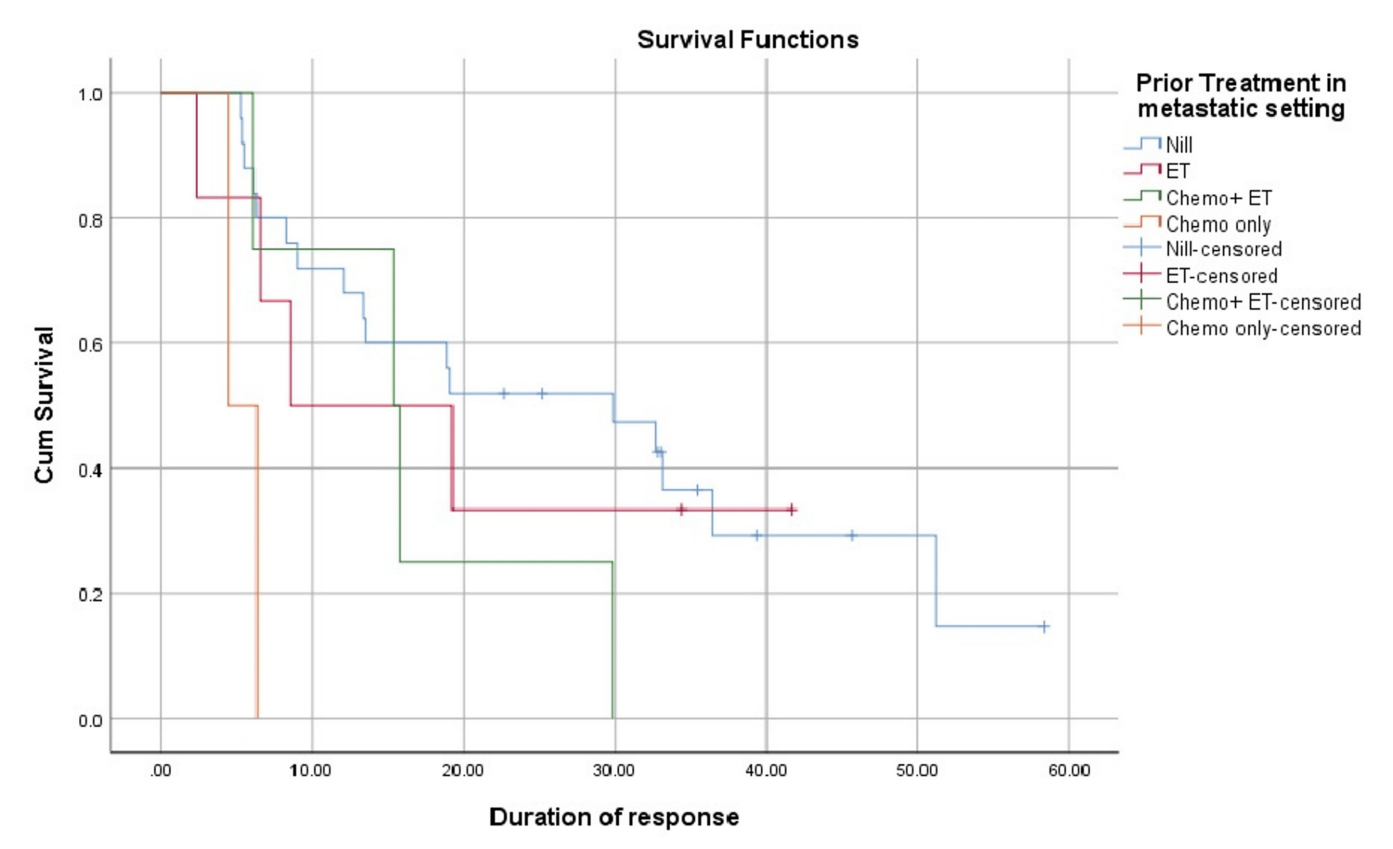
Difference in duration of response with regard to prior treatment received in a metastatic setting. ET: endocrine therapy

With regard to the endocrine agent received in combination with ribociclib (P-value 0.003), the median DOR was 51.1 months with tamoxifen (n = 4), 18.9 months with letrozole (n = 24), 6.1 months with fulvestrant (n = 8), and 19.2 months for one patient who had received exemestane. This is shown in Figure 3 and Table 2 as below.

**Table 2:** Effect of patient and disease characteristics on outcomes OS: overall survival; PFS: progression-free survival; DOR: duration of response; LN: lymph nodes; ET: endocrine therapy

Parameters	Median OS (m)	P value (OS)	Median PFS (m)	p-value (PFS)	DOR (m)	p-value (DOR)
All patients	30.8	0.18	14.7		18.9	
Metastatic presentation		0.29		0.155		0.009
Denovo/treatment naïve	33.5		20.7		29.8	
Recurrent/previously treated	29.1		14.0		8.9	
Metastatic sites		0.014		0.000		0.337
Bones only	29.1		21.9		6.1	
LN only	27.7		14.2		8.3	
Distant nodal and bones	NR		57.0		33.0	
Visceral metastases	32.7		14.7		13.5	
Brain and extracranial visceral metastases	10.4		5.0		--	
Prior treatment						
Adjuvant setting		0.45		0.13		0.04
None	32.7		20.7		19.2	
ET only	30.8		14.7		12.0	
ET+chemotherapy	24.9		10.5		6.3	
Metastatic setting		0.000		0.006		0.01
None	39.7		21.9		22.6	
ET only	63.7		12.2		8.6	
ET+chemotherapy	18.3		7.2		15.3	
Only chemotherapy	13.6		11.2		4.4	
Combination agent		0.082		0.035		0.006
Tamoxifen	-		57.0		34.4	
Letrozole	32.7		19.0		18.9	
Fulvestrant	20.9		10.2		6.1	
Exemastane	18.3		6.3		19.2	

Differences in PFS based on subgroup analysis

Median PFS was 57 months in patients who had distant nodal and bone metastatic disease (n = 11), 21.9 months in those with only bone disease (n = 5), 14.7 months in the group that had visceral disease (n = 27), and five months in the patient group that had brain metastases along with extracranial visceral disease (n = 5) (P-value 0.000). 

**Figure 4:**
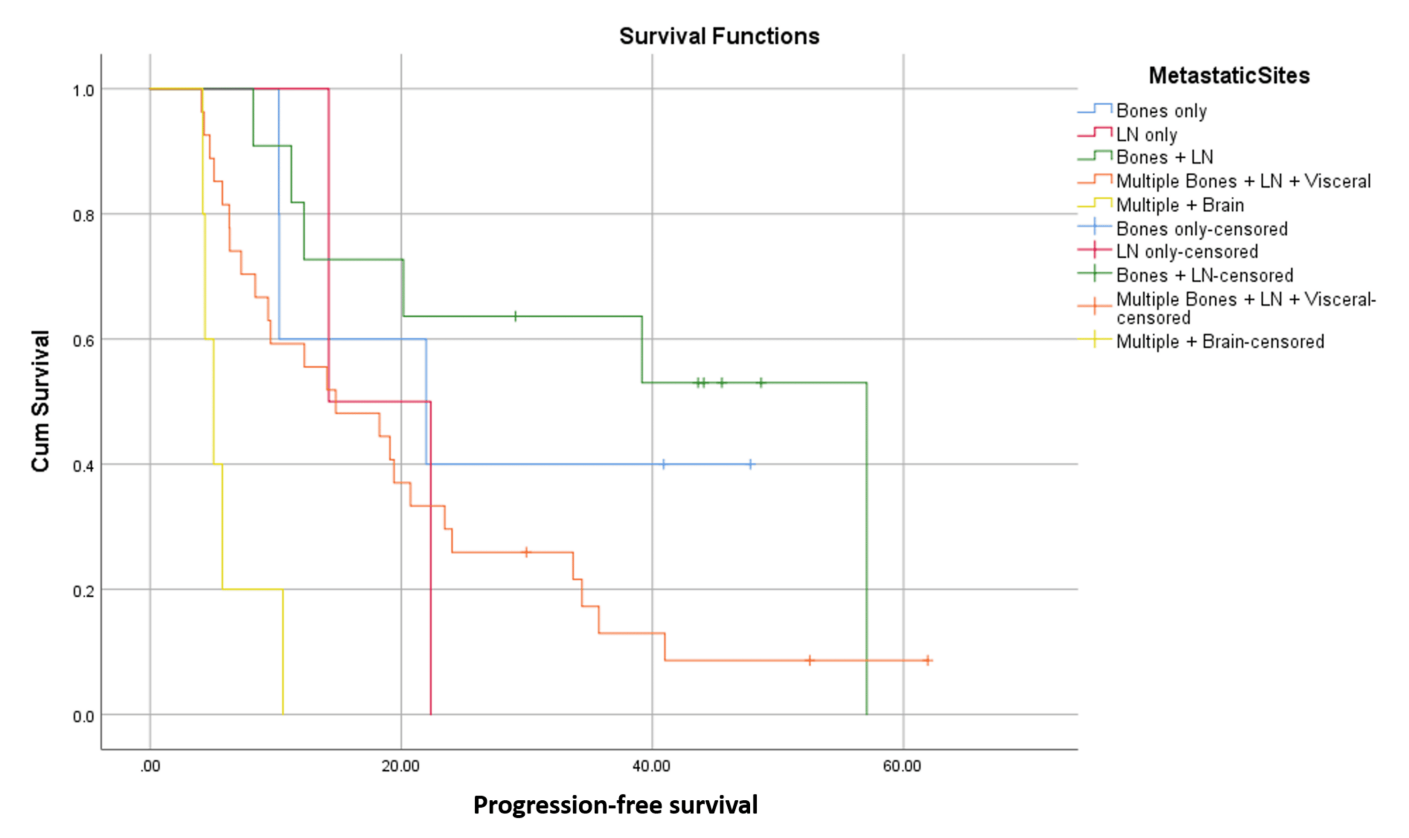
Progression-free survival in months from commencement of ribociclib therapy; differences with regard to metastatic sites. LN: lymph nodes

**Figure 5:**
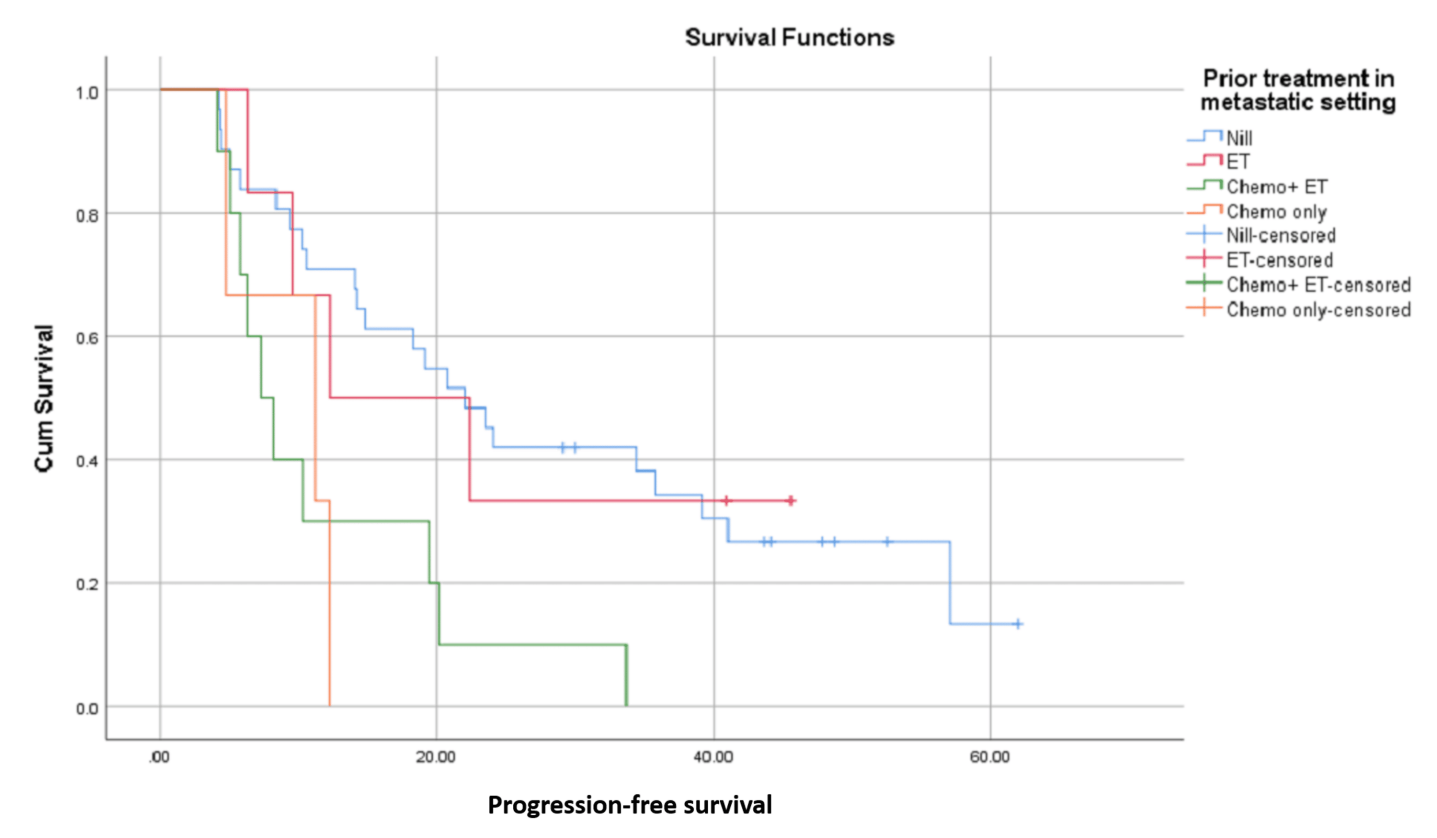
Progression-free survival in months from commencement of ribociclib therapy; differences with regard to prior treatments received in the metastatic setting. ET: endocrine therapy; chemo: chemotherapy

In another subgroup analysis, patients who had not received any prior treatment in a metastatic setting (n = 31) had a median PFS of 21.9 months, but only 12.2 months for those who had received ET only (n = 6), 11.2 for those who had only chemotherapy (n = 3), and 7.2 months for those who had both ET and chemotherapy (n = 10) (P-value 0.006). This is shown in Figures 4, 5.

Differences in OS based on subgroup analysis 

Upon subgroup analysis, median OS was “not reached” in patients who had distant nodal and bone metastatic disease (n = 11), 29.1 months in patients with only bone disease (n = 5), 32.7 months in the cohort of patients with multiple metastatic sites including visceral disease (n = 27), and 10.4 months in the group that had brain metastases along with extracranial visceral disease (n = 5) (P-value 0.14). 

**Figure 6:**
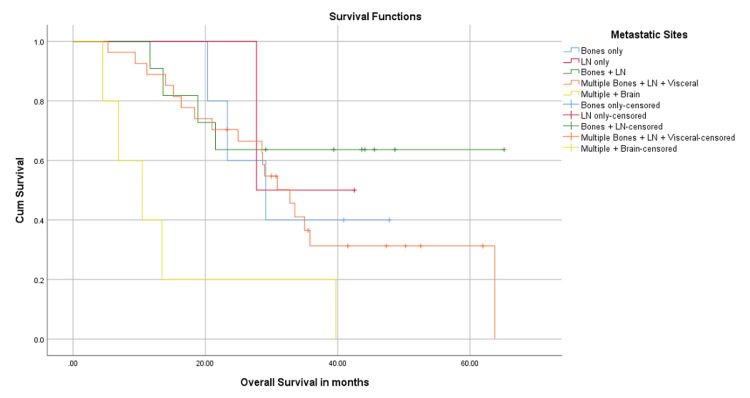
Overall survival in months; differences with regard to metastatic sites. LN: lymph nodes

**Figure 7:**
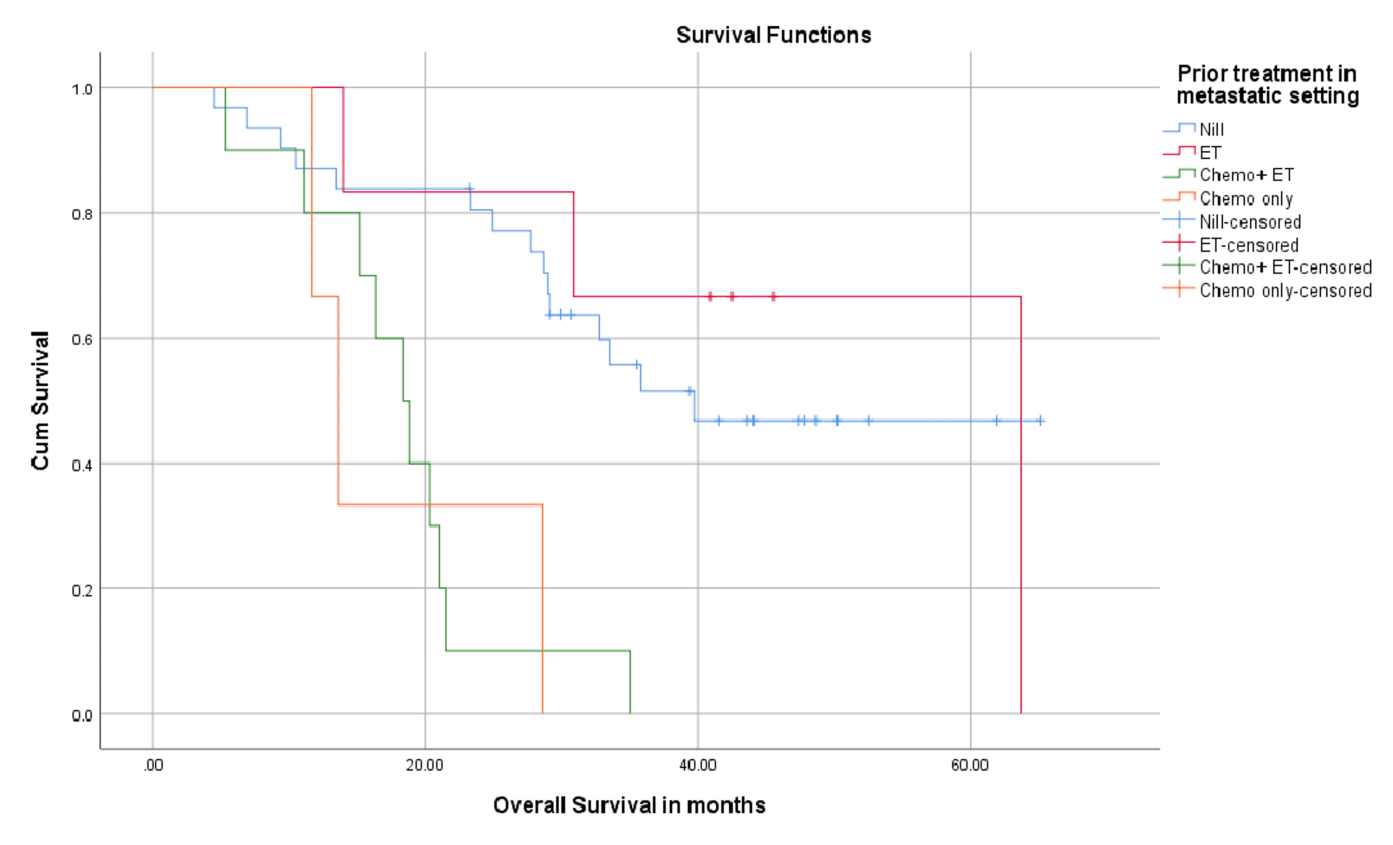
Overall survival in months; differences with regard to prior treatment in a metastatic setting. ET: endocrine therapy; chemo: chemotherapy

Patients who had not received any prior treatment in a metastatic setting (n = 31) had a median OS of 39.7 months. Those who had ET (n = 6) had a median OS of 63.7 months, those who had received ET as well as chemotherapy (n = 10) had a median OS of 18.3 months, and the group that had chemotherapy had a median OS of only 13.6 months (P-value 0.000). This is shown in Figures 6, 7.

Toxicity

During the course of their treatment, 46 (92%) patients had toxicities attributed to ribociclib. Of these, 18 (36%) had Grades 1-2, and 28 (56%) had Grades 3-4 toxicities. Neutropenia higher than Grade 2 occurred in 21 patients, of whom 14 required dose reductions, and two developed febrile neutropenia. Of nine patients who had elevations in the levels of transaminases, two patients required dose reductions, and one had to discontinue therapy. One patient required discontinuation of ribociclib due to a second occurrence of Grade 2 prolongation in QTc interval on ECG. However, it is possible that there might be improper documentation of adverse events in the patients' medical records due to underreporting. This is shown in Table 3 below.

**Table 3:** Incidence of toxicities and dose modifications in patients

	Any grade n (%)	Grades 1-2	Grades 3-4	Dose reductions
Any toxicity	46 ( 92)	18 (36)	28 (56)	25 (50)
Neutropenia	34 (68)	13 (26)	21 (42)	14 (28)
Anemia	9 (18)	6 (12)	3 (6)	4 (8)
Elevated transaminases	9 (18)	7 (14)	2 (4)	3 (6)
Gastritis	2 (4)	1 (2)	1 (2)	1 ( 2)
Prolonged QTc interval	1 (2)	1 (2)	-	1 (2)
Deranged renal functions	2 (4)	1 (2)	1 (2)	1 (2)

## Discussion

The approved first-line treatment for HR+/HER2- ABC/MBC is a CDK 4/6 inhibitor in combination with an ET partner, usually an aromatase inhibitors (AI) alone (letrozole or exemestane) in postmenopausal cases and with ovarian ablation in premenopausal cases, or a selective estrogen receptor modulator (SERM) (tamoxifen) [15-18]. The choice of a CDK 4/6 inhibitor depends on the efficacy, toxicity, and availability of the individual CDK 4/6 inhibitor. Studies have shown that ribociclib and abemaciclib lead to improved PFS as well as OS, even in patients with visceral crises [16-19]. The choice of an ET partner also depends on various factors, such as exposure to previous ET in an adjuvant or metastatic setting, the menopausal status of the patient, and patient choice. An alternative option for treatment is ET alone in cases of low-burden disease or chemotherapy in cases of visceral crises [19]. Response and tolerance to any treatment regimen may vary with a) patient factors such as age, gender, menopausal status, comorbidities, extent of disease, site(s) of metastases, or previous treatment history, or b) treatment factors such as particular CDK 4/6 inhibitors, combination ET agents, and the need for ovarian ablation as part of the treatment. 

Ribociclib has been a fairly well-studied and time-tested therapy for patients with HR+/HER2- ABC/MBC, and we have enough data regarding its efficacy and safety profile; however, there is a scarcity of studies in this context regarding South Asia. Given the low-middle-income setup and the limited availability and limited resources to generalize the treatment, there is little awareness of the outcomes and safety of CDK 4/6 inhibitors in the local population. The aim of our study was to evaluate real-life outcomes and study the safety of the drug in the local population. 

Our study population was relatively small given the resource-limited setting. The median age of our patient population was 52.50 years, which is comparable to that reported in the contemporary literature [20,21]. Nearly all of our patients belonged to the female gender, with only one being a male patient. Menopausal status was also representative of the contemporary literature, with most of the patients being postmenopausal (60%). Consistent with the contemporary literature, most of the patients had IDC on histopathology, with the exception being more frequent Grade II than Grade III patients [20]. Similar to regional literature, >50% of patients presented with de novo metastatic disease. The majority of our patients had visceral metastases (54%) as compared to the published regional literature (51.4% [20]; 4% [21]). More than 50% of patients received ribociclib as first-line treatment in a metastatic setting (52%). 

At a median follow-up of 30 months, OS and PFS were shorter in our study population than those in the published literature. Median PFS in our study population (32% at median follow-up) was better in the first-line setting than the subsequent line setting; however, the median OS (50%) was better in patients who received ribociclib in the second-line setting after ET alone (n = 6, 12% of patients). Subgroup analyses for PFS regarding metastatic sites revealed significant differences in outcomes; they were better with non-visceral disease than visceral and brain disease, consistent with regional literature [20].

The overall response rate and the clinical benefit rate in our study (48% and 76%, respectively) were comparable to those seen in the MONALEESA-2 trial (52.7% and 80%, respectively). And the DOR was better in patients who received ribociclib in a first-line setting (median DOR 29.8 months). These results, in addition to the existing data from phase III trials, suggest that patients should be challenged with ribociclib-based therapy in the first line in metastatic settings where the benefit is expected the most. There are differences in response based on partner ET; however, the numbers are too low to make any recommendations. The toxicity profile was similar to that seen in established trials, with neutropenia being the most common adverse event, which was managed with appropriate dose reductions.

However, various factors still need to be taken into consideration before comparing the results with existing literature, like retrospective design, smaller sample size, and non-ideal circumstances, like delayed access to health care and limited access to standard of care treatment. Regarding toxicity, there might be improper documentation of adverse events in the patients' medical records due to underreporting. Finally, there was no control group for proper comparison, as done in prospective studies. Despite these limitations, this study highlights the effectiveness and acceptable tolerance of ribociclib-based therapy in HR+/HER2- ABC/MBC and reinforces the need to make this treatment option available to this patient population at subsidized rates so that most of the patients in the concerned population can benefit from it. Ribociclib-based therapy should be made a priority, especially in younger or premenopausal patients who are expected to live longer. Public support groups should be formed to make this treatment more widely available.

## Conclusions

The researchers aimed to provide insights into the real-world effectiveness and safety of this treatment approach in a setting beyond controlled clinical trials. The study concludes that ribociclib is very effective and safe with an acceptable toxicity profile and thus should be considered the first treatment of choice in all patients with HR+/Her2- ABC/MBC, as is recommended in the guidelines. Efforts should be made at the government level and the public at large to make this treatment available to every patient in the first-line setting to avail the most benefit. Public support groups can be made to identify and to make arrangements for the needy population, and the government should be encouraged to arrange this drug at subsidized rates so that the public at large can benefit, especially younger patients who are expected to live longer.

The limitations of this study are its retrospective design, small sample size, and no control group. Further studies on a larger patient cohort, preferably prospective, are required to confirm and reinforce the above-mentioned facts in our local population and to determine which patient group will gain the maximum benefit from this treatment.
